# Trichostatin A inhibits the activation of Hepatic stellate cells by Increasing C/EBP-α Acetylation *in vivo* and *in vitro*

**DOI:** 10.1038/s41598-018-22662-6

**Published:** 2018-03-13

**Authors:** Di Ding, Lin-Lin Chen, Ying-Zhen Zhai, Chen-Jian Hou, Li-Li Tao, Shu-Han Lu, Jian Wu, Xiu-Ping Liu

**Affiliations:** 1 Department of Pathology, School of Basic Medical Sciences, Fudan University, Shanghai, 200032 China; 2 Department of Pathology, The Fifth People’s Hospital, Fudan University, Shanghai, 200040 China; 3 Department of Pathology, Peking University, Shenzhen Hospital, Shenzhen, 518036 China; 4 Department of Nutrition, University of California at Davis, Davis, California, USA; 5 Department of Medical Microbiology, Key Laboratory of Molecular Virology, School of Basic Medical Sciences, Fudan University, Shanghai, 200032 China; 6 Shanghai Institute of Liver Disease, Fudan University, Shanghai, 200032 China

## Abstract

Reversal of activated hepatic stellate cells (HSCs) to a quiescent state and apoptosis of activated HSCs are key elements in the reversion of hepatic fibrosis. CCAAT/enhancer binding protein α (C/EBP-α) has been shown to inhibit HSC activation and promote its apoptosis. This study aims to investigate how C/EBP-α acetylation affects the fate of activated HSCs. Effects of a histone deacetylation inhibitor trichostatin A (TSA) on HSC activation were evaluated in a mouse model of liver fibrosis caused by carbon tetrachloride (CCl_4_) intoxication. TSA was found to ameliorate CCl_4_-induced hepatic fibrosis and improve liver function through increasing the protein level and enhancing C/EBP-α acetylation in the mouse liver. C/EBP-α acetylation was determined in HSC lines in the presence or absence of TSA, and the lysine residue K276 was identified as a main acetylation site in C/EBP-α protein. C/EBP-α acetylation increased its stability and protein level, and inhibited HSC activation. The present study demonstrated that C/EBP-α acetylation increases the protein level by inhibiting its ubiquitination-mediated degradation, and may be involved in the fate of activated HSCs. Use of TSA may confer an option in minimizing hepatic fibrosis by suppressing HSC activation, a key process in the initiation and progression of hepatic fibrosis.

## Introduction

Growing clinical evidence has demonstrated that hepatic fibrosis and early cirrhosis may be reversible with effective etiology eradication^1,2^ and that the reversal of activated hepatic stellate cells (HSCs) to a quiescent state and their apoptosis are key elements to the reversion of hepatic fibrosis^3,4^. CCAAT/enhancer binding protein α (C/EBP-α) is a member of the CCAAT/enhancer binding protein family, plays a crucial role in preadipocyte maturation and cellular growth. Our previous study exhibited that C/EBP-α is involved in inhibiting HSC activation and promoting HSC apoptosis. It was found that C/EBP-α expression was decreased during activation of primary rat HSCs through culture; and that enhanced C/EBP-α expression by plasmid transfection suppressed HSC activation^5^. Our additional evidence indicated that C/EBP-α induced HSC apoptosis both *in vitro*^6^ and *in vivo*^7^; however, it did not cause any effect on hepatocytes^8^.

It is well known that posttranslational modifications, including methylation, phosphorylation, acetylation, and among others, are common in modulating protein expression and function. Methylation, phosphorylation and ubiquitination of C/EBP-α are involved in a number of physiological processes and may be involved in many diseases^9–12^; however, the physiological role of C/EBP-α acetylation has been poorly understood in HSCs, which play a critical role as an effector in hepatic fibrosis. Therefore, the present study aims to determine how the fate of activated HSCs is affected by C/EBP-α acetylation in HSC lines and an animal model of hepatic fibrosis.

We compared the amino acid sequence of C/EBP-α to those of many nonhistone proteins, such as C/EBP-β, p53, GATA1, Ku70, and FEN1, which have been reported to be acetylated at multiple sites^13–19^, and the findings demonstrated that C/EBP-α possesses some potential acetylation sites. Therefore, it is our speculation that C/EBP-α could be acetylated under various conditions. In the present study, trichostatin A (TSA), a histone deacetylase inhibitor (HDACI), was used to suppress hepatic fibrosis caused by treatment of carbon tetrachloride (CCl_4_) in mice. HSC lines and primary rat HSCs were used to dissect how C/EBP-α acetylation affected the fate of activated HSCs. Our findings suggest that deacetylation inhibitors, such as TSA, could be potential approach in suppressing hepatic fibrosis.

## Results

### TSA inhibited CCl_4_-induced mouse liver fibrosis

First, we evaluated the effects of TSA, an inhibitor of histone deacetylases I, II and IV^20–22^, in an animal model of liver fibrosis. Mice were treated by CCl_4_ injections for 10 weeks to develop progressive liver fibrosis^8^. We used a scoring system for the determination of fibrosis/cirrhosis described by Ishak^23^ that ranges from 1 (minimal fibrosis) to 6 (cirrhosis) (See Supplementary Table S1). CCl_4_ intoxication for 10 weeks caused fibrosis, with a median Ishak score of 4.0 (interquartile range [IRE] 4.0–5.0; See Supplementary Fig. S1).

**Figure 1:**
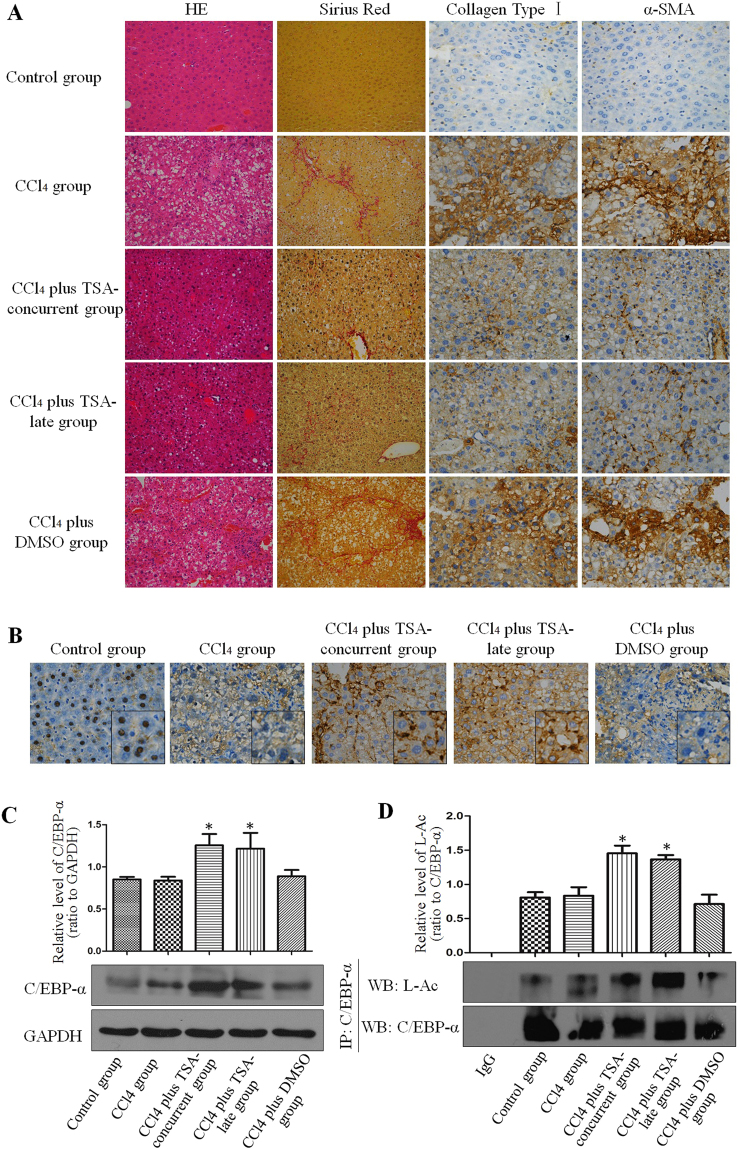
Trichostatin A inhibited CCl_4_-induced mouse liver fibrosis. (**A**) TSA ameliorates hepatic fibrosis in mice treated with CCl_4_. The significant decrease in the amount of extracellular matrix was confirmed by hematoxylin-eosin (magnification, x200) and Sirius red staining (magnification, x200) after TSA treatment. Immunohistochemical staining for α-SMA (magnification, x400) and collagen type I (magnification, x400) was performed on liver tissue samples isolated from mice in different groups. **(B)** C/EBP-α expression in the livers of mice in different groups by immunohistochemistry (magnification, x400). **(C)** Western blot showed that C/EBP-α expression in the livers of mice in different groups. *p < 0.05 compared to CCl_4_ injection group. **(D)** Western blot showed that C/EBP-α immunoprecipitated from the livers of mice in different groups was acetylated and acetylation levels of C/EBP-α were different among groups. *p < 0.05 compared to CCl_4_ injection group.

Treatment with TSA (1 mg/kg) beginning at 1st week or 6th weeks inhibited fibrogenesis as indicated by Sirius red staining and immunohistochemical staining for collagen type I (Fig. 1A). Compared to the control group, the positive area of Sirius red staining was clearly identifiable in the CCl_4_ group (3.84 ± 0.48%) and in the CCl_4_ plus DMSO group (3.6 ± 0.55%); however, it was less positive in the CCl_4_ plus TSA-concurrent group (1.34 ± 0.28%) and in the CCl_4_ plus TSA-late group (1.47 ± 0.41%). Immunohistochemical staining for collagen type I demonstrated that deposition of extracellular matrix (ECM) components was significantly reduced in the CCl_4_ plus TSA-concurrent group and CCl_4_ plus TSA-late group (2.45 ± 0.4 and 3.1 ± 0.43%, respectively) compared to CCl_4_ or CCl_4_ plus DMSO groups (6.56 ± 0.47 and 6.56 ± 0.83%, respectively). However, no significant difference between the CCl_4_ plus TSA-concurrent group and CCl_4_ plus TSA-late group was detected (Table 1). Consistent with these results, the CCl_4_ plus TSA-concurrent group and CCl_4_ plus TSA-late group had lower median Ishak semi-quantitative scores of 1.0 (IQR 1.0–2.0) and 2.0 (IQR 1.0–3.0) respectively compared to the CCl_4_ group (median Ishak score 4.0, IQR 4.0–5.0) and CCl_4_ plus DMSO group (median Ishak score 4.0, IQR 3.0–4.5).

**Table 1:** Hydroxyproline content, sirius red staining, collagen type I and α-SMA in different groups. Values represent means ± SD (n = 6 each group). *p < 0.05 versus control group, ^#^*p* < 0.05 versus CCl_4_ group, ^&^*p* < 0.05 versus CCl_4_ plus TSA-concurrent group. CCl_4_ = carbon tetrachloride; TSA = trichostatin A; DMSO = dimethyl sulfoxide.

Groups	Hydroxyproline content (μg/mg)	Sirius red staining (%)	Collagen type I positive area (%)	α-SMA positive area (%)
Control group	0.03 ± 0.007	0.53 ± 0.18	1.1 ± 0.06	1.1 ± 0.17
CCl_4_ group	0.15 ± 0.027*	3.84 ± 0.48*	6.56 ± 0.47*	6.68 ± 0.4*
CCl_4_ plus TSA-concurrent group	0.09 ± 0.007*^#^	1.34 ± 0.28*^#^	2.45 ± 0.4*^#^	2.68 ± 0.28*^#^
CCl_4_ plus TSA-late group	0.11 ± 0.006*	1.47 ± 0.41*^#^	3.1 ± 0.43*^#^	3.34 ± 0.35*^#&^
CCl_4_ plus DMSO group	0.17 ± 0.048*	3.6 ± 0.55*	6.56 ± 0.83*	6.18 ± 0.54*

The hydroxyproline content in the control group was 0.03 ± 0.007 μg/mg. The CCl_4_ group demonstrated a dramatic elevation in the hydroxyproline content (0.15 ± 0.027 μg/mg). The hydroxyproline content was decreased in the CCl_4_ plus TSA-concurrent group (0.09 ± 0.007 μg/mg). However, no significant difference was noted between the CCl_4_ plus TSA-late group and the CCl_4_ group (Table 1). These results are consistent with Sirius red staining and immunohistochemical staining for collagen type I.

HSC activation was observed by immunohistochemical staining for α-smooth muscle actin (α-SMA), which is a marker of activated HSCs^24,25^. Few α-SMA-positive cells were primarily located in the vasculature of normal liver tissue; however many α-SMA-positive cells were observed at fibrotic septa and the vasculature in the liver tissue of CCl_4_ group mice. Weaker α-SMA staining was observed in incomplete septa in the CCl_4_ plus TSA-concurrent group and in the CCl_4_ plus TSA-late group. The number of α-SMA-positive HSCs was significantly reduced in the CCl_4_ plus TSA-concurrent group and CCl_4_ plus TSA-late group compared to the CCl_4_ group and CCl_4_ plus DMSO group (Fig. 1A; Table 1).

Liver biochemical parameters as shown in Table 2, demonstrated that there was significant improvement in liver injury with concurrent and late TSA intervention as indicated by much lower levels of alanine aminotransferase (ALT), γ-Glutamyltranspeptidase (γ-GT) and alkaline phosphatase (AKP) compared to the CCl_4_ group. These findings suggested that TSA treatment ameliorated CCl_4_-induced hepatic fibrosis in combination with improved injury levels.

**Table 2:** Effect of TSA on sera biochemical assays of total protein, albumin, AST, ALT, γ-GT and AKP. Values represent means ± SD (n = 6 each group). *p < 0.05 versus control group, #p < 0.05 versus CCl4 group, ^&^p < 0.05 versus CCl4 plus TSA-concurrent group. AST = aspartate amino transferase; ALT = alanine aminotransferase, γ-GT = γ-Glutamyltranspeptidase, AKP = alkaline phosphatase.

Groups	Total protein (g/L)	Albumin (g/L)	AST (U/L)	ALT (U/L)	γ-GT (U/L)	AKP (U/L)
Control group	53.44 ± 3.25	23.39 ± 1.24	11.21 ± 0.93	9.88 ± 1.3	2.71 ± 1.06	103.79 ± 11.66
CCl_4_ group	48.79 ± 2.87*	30.38 ± 0.49*	28.03 ± 5.78*	34.77 ± 18.58*	6.76 ± 1.69*	339.1 ± 21.91*
CCl_4_ plus TSA-concurrent group	48.3 ± 4.13*	27.67 ± 2.26*	18.65 ± 1.81*	10.66 ± 0.83^#^	4.37 ± 0.64*^,#^	274.09 ± 19.77*^,#^
CCl_4_ plus TSA-late group	49.67 ± 2.25*	29.37 ± 2.9*	17.88 ± 0.9*	10.37 ± 0.62^#^	4.01 ± 1.01^#^	295.08 ± 9.24*^,#,&^
CCl_4_ plus DMSO group	51.74 ± 3.35	30.7 ± 2.61*	26.29 ± 3.26*	33.39 ± 12.31*	6.7 ± 1.86*	367.5 ± 16.96*

Next, we examined the C/EBP-α protein level of mouse livers in different groups. Immunohistochemical staining showed that C/EBP-α was primarily expressed in hepatocyte nuclei in control livers; whereas, its expression was greatly reduced after long-term CCl_4_ intoxication (CCl_4_ or CCl_4_ plus DMSO groups). C/EBP-α expression increased following TSA injections (Fig. 1B). Co-immunofluorescent staining of C/EBP-α with α-SMA in livers from mice treated with CCl_4_ plus TSA group showed the co-localization of C/EBP-α in nucleus and α-SMA in cytosol of hepatic stellate cells. (Supplementary Fig. S2). The result of Western blot analysis is consistent with the result of C/EBP-α immunohistochemical staining (Fig. 1C). Then we performed immunoprecipitation using anti-C/EBP-α antibody to pull down the protein from liver lysates, and then performed Western blot analysis with anti-acetyllysine antibody. Interestingly, as shown in Fig. 1D, the acetylation level of C/EBP-α was enhanced in the presence of inhibitor of histone deacetylases. This result implies that acetylation may exist in C/EBP-α protein isolated from mouse liver.

### C/EBP-α is acetylated in HSCs and its main acetylation site is the residue K276

**Figure 2:**
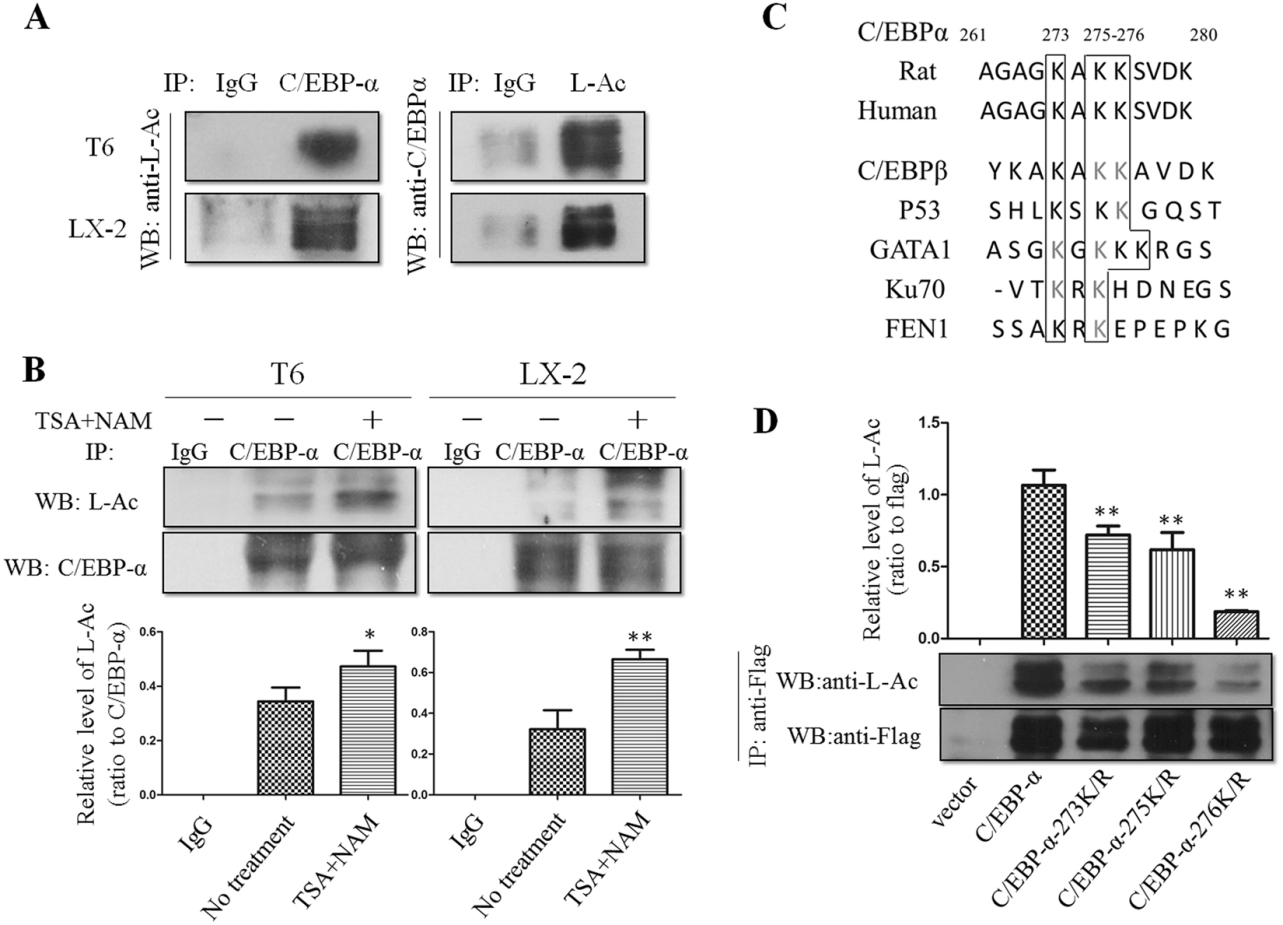
C/EBP-α is acetylated in HSCs and its main acetylation site is the residue K276. (**A**) Western blot showed that C/EBP-α immunoprecipitated from HSC-T6 and LX-2 cell lysates was acetylated. **(B)** Acetylation of C/EBP-α was enhanced in the presence of TSA and NAM. *p < 0.05 and **p < 0.01 compared to no treatment group. **(C)** Sequence alignment of a potential acetylation site in C/EBP-α and other proteins as indicated. **(D)** Mutation of K276 significantly decreased C/EBP-α acetylation. The indicated plasmids were transfected into 293 T cells, and proteins were immunoprecipitated for Western blot. **p < 0.01 compared to C/EBP-α group.

To identify whether C/EBP-α is acetylated in HSC-T6 and LX-2 cells, we performed immunoprecipitation using anti-C/EBP-α antibody to pull down the protein from HSC-T6 and LX-2 cell lysates, and then performed Western blot analysis with anti-acetyllysine antibody. As shown in Fig. 2A, specific C/EBP-α protein from lysates of these two HSC lines was pulled down by anti-C/EBP-α antibody. The immune-precipitated protein was analyzed by Western blotting with anti-acetyllysine antibody. These results indicated that there was acetylation in C/EBP-α protein at lysine residues in partially activated HSC lines. In addition, we treated HSCs with TSA (500 nM) and nicotinamide (NAM, 5 mM; an inhibitor of histone deacetylase III, which is also known as the SIRT family of deacetylases)^20–22^, and it appeared that acetylation of C/EBP-α was enhanced in the presence of these inhibitors (Fig. 2B). This result confirmed our initial finding that C/EBP-α could be acetylated during HSC activation.

To compare the amino acid sequence of C/EBP-α with those of many nonhistone proteins that have been reported to be acetylated at multiple sites, such as C/EBP-β, p53, GATA1, Ku70 and FEN1, we determined a pattern of potential acetylation sites (Fig. 2C). Using the pattern, we predicted that lysine residues at 273, 275, and 276 of C/EBP-α were possible acetylation sites. To confirm this prediction, we mutated each of these three lysine residues of C/EBP-α individually to arginine (R), and examined their acetylation in transfected 293T cells. Western blot analysis showed that arginine substitution at the K276 residue, but not at K273 or K275 residues, resulted in a significant reduction in C/EBP-α acetylation (Fig. 2D). This result confirmed that K276 is a major acetylation site in C/EBP-α.

### Acetylation of C/EBP-α increases its stability and protein level by inhibiting ubiquitination

**Figure 3:**
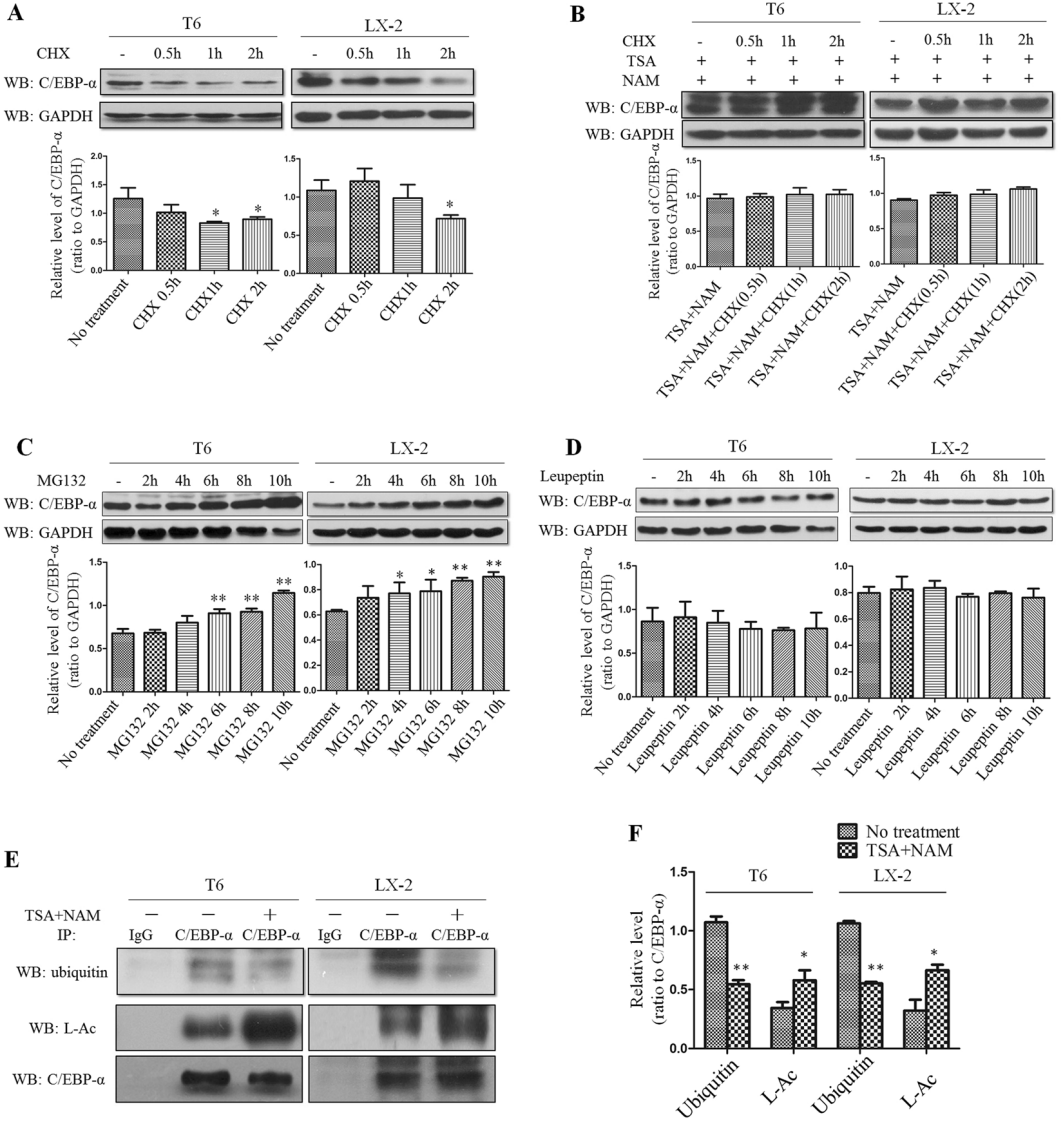
Acetylation of C/EBP-α increases its stability and protein level by inhibiting ubiquitination. (**A**) CHX treatment leads to a time-dependent reduction in C/EBP-α as determined by Western blot analysis. **(B)** TSA and NAM treatment blocks the effect of CHX on C/EBP-α protein levels. **(C)** The ubiquitin proteasome inhibitor MG132 causes the accumulation of C/EBP-α protein. **(D)** The lysosomal proteasome inhibitor leupeptin had no effects on C/EBP-α protein. **(E** and **F)** Deacetylase inhibitors promote the acetylation of C/EBP-α but inhibit ubiquitination. *p < 0.05 and **p < 0.01 compared to no treatment group.

To determine the effect of acetylation on C/EBP-α, we examined the stability of the C/EBP-α protein after treatment with cycloheximide (CHX, 200 μg/ml) alone or in combination with TSA (500 nM) and NAM (5 mM). Because CHX suppresses protein synthesis by interfering translocation, the protein level will be decreased with time due to reduced synthesis. It was evident that CHX treatment led to a time-dependent reduction in C/EBP-α content in HSC-T6 and LX-2 cells as determined by Western blot analysis (Fig. 3A). However, this reduction was abrogated when cells treated with TSA and NAM in the presence of CHX (Fig. 3B). These results indicated that the histone deacetylase inhibitors, such as TSA and NAM, maintained the protein level of C/EBP-α. Therefore, it appeared that the maintenance of C/EBP-α protein levels by TSA or NAM was achieved through their role in enhancing acetylation as demonstrated in Fig. 2B.

To further prove the effect of acetylation on C/EBP-α stability, we investigated dynamic changes between acetylation and the degradation of C/EBP-α. First, to determine the degradation pathway for C/EBP-α, we treated cells with MG132 (10 μM), which is a proteasome inhibitor that blocks ubiquitin-proteasome protein degradation. This treatment caused a significant accumulation of C/EBP-α protein (Fig. 3C). In contrast, treatment with alysosomal protease inhibitor, leupeptin (50 μM), did not increase C/EBP-α protein level (Fig. 3D**)**. These results confirmed the involvement of the ubiquitin-proteasome system in C/EBP-α degradation. The ubiquitin-proteasome system mediates protein degradation through ubiquitination. Next, we performed immunoprecipitation using anti-C/EBP-α antibody to pull down C/EBP-α from HSCs treated with TSA and NAM. C/EBP-α acetylation and ubiquitination were then analyzed by Western blot analysis with the indicated antibodies. As shown in Fig. 3E and F, the acetylation level of C/EBP-α increased after treatment with TSA and NAM; whereas the level of ubiquitin linked to C/EBP-α decreased after the same treatment. Based on these results, it is conclusive that TSA and NAM promoted the acetylation of C/EBP-α, resulting in inhibition of C/EBP-α ubiquitination. This inhibition led to decreased ubiquitination-mediated C/EBP-α degradation. Therefore, the C/EBP-α protein level increased as result of C/EBP-α protein accumulation after treatment with HDACIs, such as TSA and NAM.

### TSA treatment increased C/EBP-α and suppressed HSC activation through enhanced apoptosis

**Figure 4:**
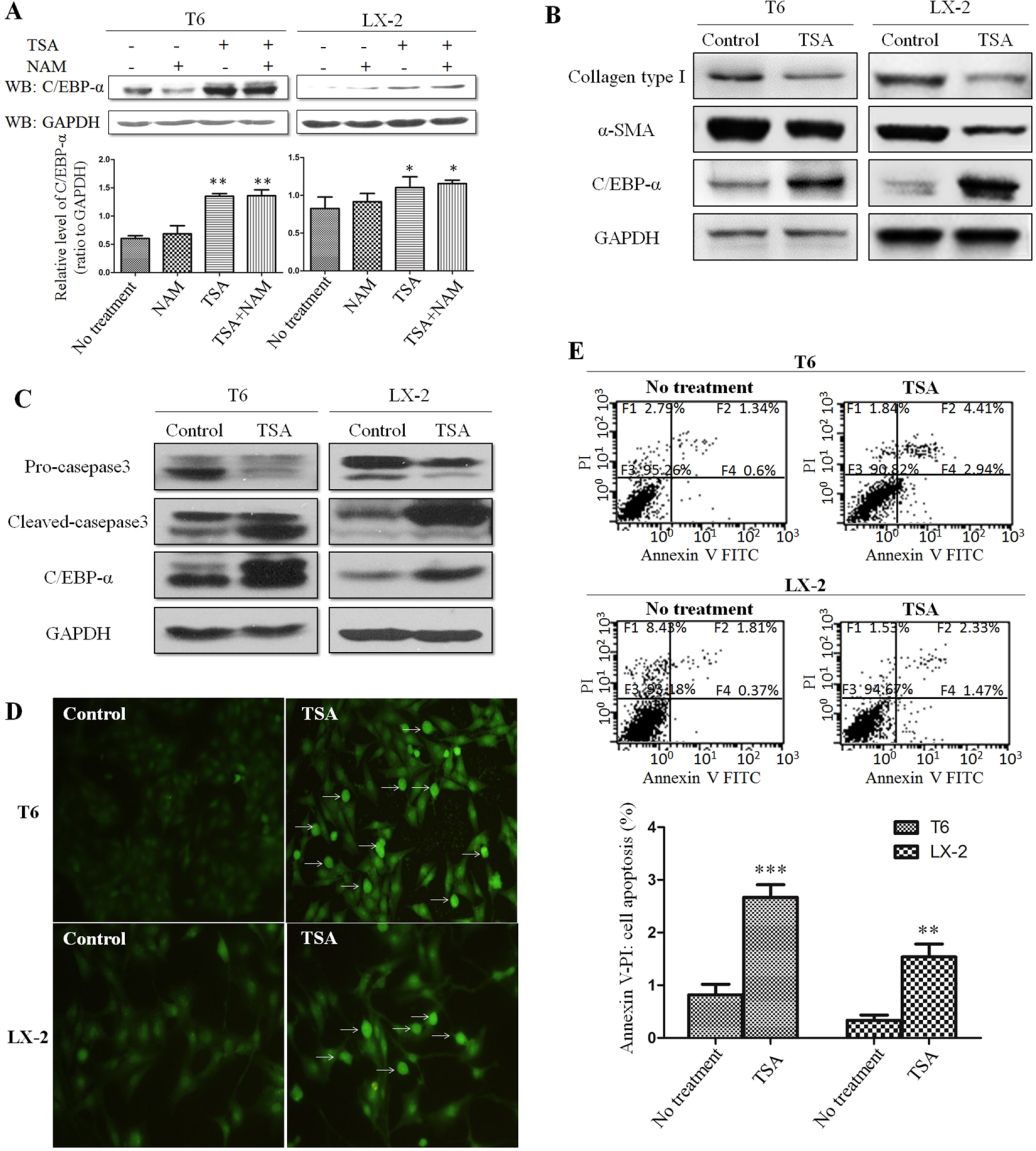
TSA treatment increased C/EBP-α and suppressed HSC activation through enhanced apoptosis. (**A**) TSA treatment caused the accumulation of C/EBP-α protein. **(B)** The level of C/EBP-α, α-SMA and collagen type I were detected by Western blot analysis in cells treated with TSA. **(C)** Expression of caspase-3 and cleaved-caspase-3 in cells after treatment with TSA. **(D)** Different levels of apoptosis were detected by TUNEL in cells untreated or treated with TSA. **(E)** Different ratios of apoptosis were detected by annexin V/PI flow cytometry in cells untreated or treated with TSA. **p < 0.01 and ***p < 0.001 compared to no treatment group.

To further confirm the effect of HDACIs on C/EBP-α role in HSC activation, HSC-T6 and LX-2 cells were treated with TSA or NAM, and C/EBP-α protein levels were determined by Western blot analysis. As expected, C/EBP-α protein level was increased by TSA treatment; whereas NAM did not affect C/EBP-α protein level significantly (Fig. 4A). Thus, these findings confirmed that TSA increased C/EBP-α protein levels and suppressed activity of deacetylase that participates in the acetylation of C/EBP-α. This might confer a new approach in blocking HSC activation.

Enhanced α-SMA and collagen type I levels are considered to be the valuable markers for HSC activation. As shown in Fig. 4B, while level of C/EBP-α was increased, levels of α-SMA and collagen type I were significantly decreased by TSA treatment.

To further understand the underlying mechanism of decreased HSC activation caused by TSA, activation of caspase-3, an important marker of apoptosis, was determined in HSC-T6 and LX-2 cells under TSA treatment. As shown in Fig. 4C, cleaved caspase-3 levels were increased in HSC-T6 and LX-2 cells after TSA treatment; whereas, the pro-caspase-3 protein level was decreased. We also assayed activity of both caspase-8 and caspase-9. The results indicated that activity of these molecules was also increased; however, caspase-9 activity did not reach statistical significance (See Supplementary Fig. S3). Next, both terminal deoxynucleotidyltransferase (TdT)-mediated dUTP nick end-labeling (TUNEL) and annexin V/PI flow cytometric assays were employed to verify apoptotic extent of HSC-T6 and LX-2 cells after TSA treatment. It was clear that TSA treatment promoted apoptosis in both HSC-T6 and LX-2 cells (Fig. 4D and E). Taken together, it is evident that TSA treatment suppressed HSC activation largely through promoting apoptosis, in which acetylation may be critical for this process.

### TSA suppressed activation of primary rat HSCs

**Figure 5:**
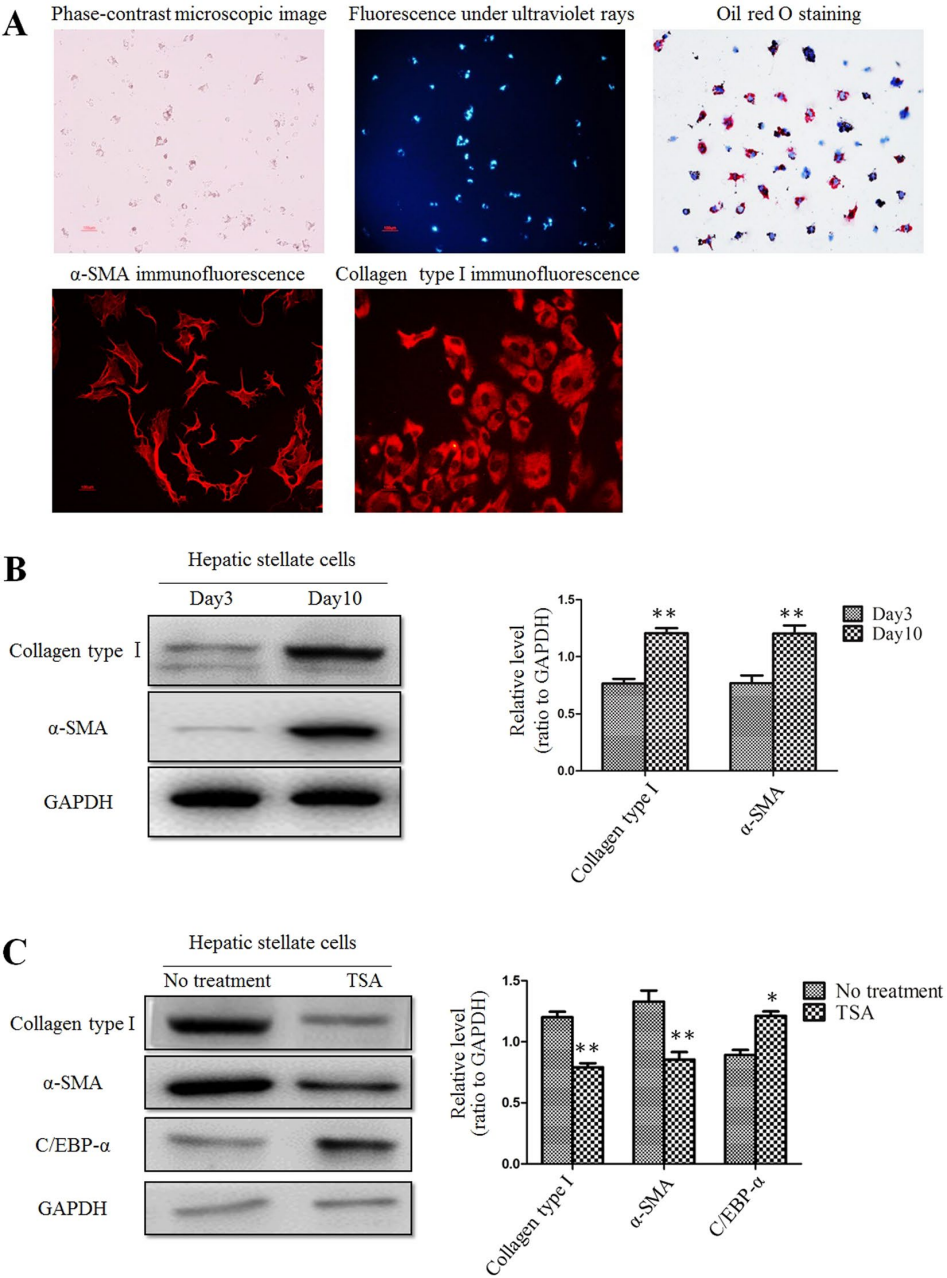
TSA suppressed activation of primary rat HSCs. (**A**) Primary cultured rat HSCs were auto-activated during culture. This was confirmed by fluorescence image, immunofluorescent staining and oil red O staining. **(B)** Western blot analysis showed that the protein levels of collagen type I and α-SMA in day 10 HSCs were increased compared to day 3 cells. **p < 0.01 compared to day 3 group. **(C)** Expression of collagen type I, α-SMA and C/EBP-α in day 10 HSCs after treatment with TSA. *p < 0.05 and **p < 0.01 compared to no treatment group.

In order to verify whether TSA has the same effects in primary HSCs, rat primary HSCs were used in the same setting of experiments. Fluorescent images and oil red O staining confirmed that HSCs with sufficient purity and viability were successfully isolated from rat liver. Primary cultured rat HSCs were auto-activated during culture, a finding that was confirmed by staining with α-SMA, a marker of activated HSCs^24,25^ (Fig. 5A). α-SMA expression in HSCs cultured up to 10 days was significantly higher than those cultured up to 3 days. Collagen type I expression in primary HSCs was also increased on Day 10 (Fig. 5B). Next, we treated primary HSCs at day 10 with TSA for 16 hours, and then examined C/EBP-α protein level. As shown in Fig. 5C, C/EBP-α protein levels were increased significantly; whereas, expression of α-SMA and collagen type I was decreased after TSA treatment, implying that deacetylation in primary HSCs suppressed HSC activation probably through the increasing C/EBP-α protein levels.

## Discussion

HSC activation plays a pivotal role in the advance of liver fibrosis, and these cells represent an appealing target for anti-fibrotic therapy. Our previous studies^5–8^ showed that an exogenous increase C/EBP-α expression not only suppressed HSC activation, but also induced an apoptotic fate of activated HSCs. In this study, we demonstrated that the status of C/EBP-α acetylation affects the fate of activated HSCs as indicated by the fact that TSA, an HDACI, increased endogenous C/EBP-α protein levels. Our work also confirmed that C/EBP-α is acetylated majorly at the residue K276 site.

Acetylation is a reversible and highly controlled process of post-transcriptional modification that is primarily observed on lysine residues. In 1997, acetylation of the first nonhistone protein, p53, was reported; that the findings documented that acetylation enhanced p53 DNA-binding activity^14^. Since then, more nonhistone proteins, such as C/EBP-β, GATA1, Ku70, FEN1, E2F1 and EKLF, have been found to be acetylated^13,17–19,26,27^. C/EBP-α has been reported to interact with Tip60, which is a member of the MYST acetyltransferase family^28^. Our results showed that C/EBP-α expressed in HSCs is acetylated, and that residue K276 is the main acetylation site of C/EBP-α. However, further studies remain to be conducted to investigate the impact of acetylation of different residues in C/EBP-α, as there are a total of 15 lysine residues in the amino acid sequence of C/EBP-α^29,30^.

C/EBP-α protein consists of an activation domain, a DNA-binding basic region, and a leucine zipper domain^31^. The lysine residue K276 which was identified as a main acetylation site of C/EBP-α protein in this study is located in the DNA-binding basic region of C/EBP-α. The rat C/EBP-α gene shows almost complete sequence identity with the mouse. The promoter regions of the human, chicken and xenopus C/EBP-α genes show only limited sequence identity with the mouse/rat promoter (e.g. 53% identity in the case of the human promoter)^32^. However, C/EBP-α shares the highly conserved DNA-binding basic region and leucine zipper domain that are involved in dimerization and DNA binding^31,33^. Therefore, presumably, there is less possibility that difference exists in the lysine residue K276 identified as a main acetylation site of C/EBP-α protein between different species. That’s the issue we will look into in the future studies.

In the present study, TSA and NAM were used to suppress deacetylase activity as potent HDACIs. TSA inhibits deacetylase that functions with zinc as a coenzyme; whereas, NAM inhibits the deacetylase that functions with NAD+ as a coenzyme^20–22^. Using both inhibitors, all types of deacetylases in cells should be completely suppressed. Therefore, as expected, C/EBP-α acetylation extent was increased after the treatment of TSA and NAM in HSCs (Fig. 2B). As shown in Fig. 4A, TSA appears to be more effective than NAM for C/EBP-α stability. This finding indicated that the deacetylase that participates in the acetylation of C/EBP-α might belong to deacetylase functioned with zinc as a coenzyme.

In the present study, C/EBP-α protein levels were increased in HSCs after treatment with HDACIs. It is well known that protein levels are dependent on a dynamic balance between protein synthesis and degradation in a cell. Therefore, after HSC-T6 and LX-2 cells were treated with CHX, a time-dependent reduction in C/EBP-α protein levels was observed, indicating that C/EBP-α protein synthesis was suppressed by CHX. Then, both CHX and HDACIs were used to treat HSC-T6 and LX-2 cells, and the results showed that the C/EBP-α protein levels were maintained. This finding indicated that the HDACIs counteracted the role of CHX, and maintained C/EBP-α protein. Next, MG132, an inhibitor of the ubiquitin-proteasome protein degradation pathway, was found to increase the C/EBP-α protein level by inhibiting C/EBP-α degradation. Therefore, we postulated that C/EBP-α is degraded through the ubiquitination pathway, and the results of the experiment with a lysosomal protease inhibitor (leupeptin) supported this speculation. More interestingly, it is evident that C/EBP-α acetylation was increased after treatment with HDACIs; whereas the ubiquitination of C/EBP-α was decreased as demonstrated by immunoprecipitation and Western blotting. These results indicated that there exists a competitive inhibition between acetylation and ubiquitination in the C/EBP-α protein. Therefore, it is our assumption that acetylation inhibits C/EBP-α ubiquitination; in turn results in an increase in C/EBP-α protein level as a result of decreased ubiquitination-mediated degradation. Thus, it is conceivable that HDACIs increased C/EBP-α protein levels through enhancing its stability. In this aspect, TSA appears to be more effective than NAM for C/EBP-α stability (Fig. 4A).

The supplementary Figure S2 showed that C/EBP-α was mainly located in nucleus of HSCs. And our previous study demonstrated that inherent C/EBP-α protein was increased primarily in the nucleus of HSC-T6 cells following treatment with TSA^34^. These results further verified that C/EBP-α predominantly functions in the nucleus of HSCs.

HSC activation is the process in which HSCs transform from star-like lipocytes in a quiescent state into myofibroblast-like cells (MFs)^35,36^. Activated HSCs are the major source of extracellular matrix (ECM) components, including collagen type I, III; and α-SMA expression is a typical characteristic of activated HSCs. It is commonly seen that C/EBP-α protein level is decreased during HSC activation^5^; whereas α-SMA and collagen type I levels are increased remarkably^24,25^. In our previous study, C/EBP-α protein level was increased in activated HSCs by non-physiologic approaches, such as plasmid transfection and viral infection; and the exogenous expressed C/EBP-α has been shown to inhibit HSC activation and displayed a repressing effect on hepatic fibrosis^6–8^. On the other hand, an increase in the C/EBP-α protein level could be achieved by suppressing the ubiquitination-mediated degradation of C/EBP-α, as demonstrated in the present study. It is intriguing that treatment with TSA caused an increase in C/EBP-α protein level, while protein levels of α-SMA and collagen type I were decreased in these HSCs, indicating that treatment with TSA may actually lead MFs to revert into a quiescent state of star-like lipocytes. Our findings are in dose consistence with the report by Toshiro Niki *et al*.^37^. Moreover, TSA was found to promote the apoptosis of HSC-T6 and LX-2 cells, a promising fate of activated HSC, mainly through the activation of caspase pathway. Taken together, these findings indicated that C/EBP-α acetylation plays a key role in the reversal of liver fibrosis as demonstrated in both HSC cell lines and primary cultured HSCs in the present study.

The effects of TSA *in vitro* experiments were consistent with the results obtained from the animal model of liver fibrosis. Repeated injection of CCl_4_ in mice led to progressive liver fibrosis. C/EBP-α expression was greatly reduced in the CCl_4_-treated mice compared to the controls; however its expression was increased by the injection of TSA, probably through enhancing C/EBP-α acetylation (Fig. 1D). When TSA was used in animals, it was not selectively delivered to HSCs, and therefore other cell types, such as hepatocytes were exposed to it. Although C/EBP-α protein was shown in both fibrotic septa and non-fibrotic region, Fig. 1B demonstrates that C/EBP-α expression was obviously enhanced by the treatment of TSA in the fibrotic septa where the activated HSCs are primarily located in. In addition, findings from our previous study demonstrated that HSC-T6 cells were more sensitive than BRL-3A hepatocytes to TSA treatment^34^.

Less positivity in α-SMA and collagen type I staining in incomplete septa in the group with TSA intervention indicated that TSA was effective in suppressing HSC activation in CCl_4_-treated mice. Sirius red staining and hydroxyproline quantitation documented that hepatic fibrosis was alleviated with TSA intervention compared to CCl_4_-treated mice. Moreover, liver damage markers, such as ALT, γ-GT and AKP, were decreased in mice with TSA intervention, confirming that C/EBP-α acetylation is beneficial for the reversal of liver fibrosis through attenuating liver injury.

In conclusion, the findings in the present study provide the evidence that C/EBP-α is acetylated in HSCs. Increased acetylation of C/EBP-α by TSA may inhibit HSC activation by an increase in C/EBP-α protein level through enhancing its stability. Importantly, reducing liver injury by TSA may contribute to its effects on ameliorating liver fibrosis in addition to its direct effects on reversal of activated HSCs to a quiescent state. Taken together, this study confers a novel pharmacological intervention approach in suppressing hepatic fibrosis.

## Materials and Methods

### CCl_4_-induced mice fibrosis model and TSA injection ***in vivo***

Male Balb/c mice (7–10 weeks old; body weight, 18–20 g) were obtained from the Chinese Academy of Sciences (Shanghai), housed in one of the facilities of the University Animal Center. Animal handling was performed in accordance with the NIH Guidelines for the Care and Use of Laboratory Animals, and the protocol was approved by Animal Ethic Committee of Fudan University School of Basic Medical Sciences. Animal numbers and treatment of different groups were detailed in Table 3. Mice in the CCl_4_ group (fibrosis model) only received 25% CCl_4_ in olive oil by the repeated intraperitoneal injection twice weekly for up to 10 weeks. Mice in the CCl_4_ plus TSA group were treated with TSA (1 mg/kg, twice weekly; V900931, Sigma-Aldrich) began at the 1st or 6th weeks. At the end of the 10th week, all mice were sacrificed by exsanguination under anesthesia. The livers were taken out, fixed in 10% neutralized formaldehyde or stored at −70 °C for the following experiments. Serum samples were collected for analysis of liver biochemical parameters.

**Table 3:** Treatment of groups. CCl4 = carbon tetrachloride; TSA = trichostatin A; DMSO = dimethyl sulfoxide.

Groups	Treatment
Control group	Normal mice with no treatment
CCl_4_ group	Mice received 0.1 ml of 25% CCl_4_ dissolved in olive oil by repeated intraperitoneal injection twice a week for up to 10 weeks
CCl_4_ plus TSA-concurrent group	Mice were administrated with TSA (1 mg/kg, 30 μl, began at week 1 for concurrent intervention) by intraperitoneal injection after CCl_4_ injection
CCl_4_ plus TSA-late group	Mice were administrated with TSA (1 mg/kg, 30 μl, began at week 6 for late intervention) by intraperitoneal injection after CCl_4_ injection
CCl_4_ plus DMSO group	Mice were administrated with DMSO (30 μl) by intraperitoneal injection after CCl_4_ injection

### Sirius red staining and immunohistochemistry

For sirius red staining, sections were prepared with 5 μm thickness and deparaffinzed. Briefly, sections were incubated with 0.1% (w/v) Sirius red for 1 hour in a saturated aqueous solution (1.2% w/v) of picric acid, with a final pH of 2.0. After staining, sections were dehydrated in alcohol and washed with xylene. For immunohistochemistry, sections were prepared with 5 μm thickness and deparaffinzed. Sections were incubated in xylene 3 times for 5 min each, washed with 100% ethanol and 95% ethanol for 3 min each, and washed with dH_2_O 3 times for 3 min each. Antigen unmasking was performed in boiled 0.01 M citrate buffer (pH 6.0) for 20 min. After cooling at RT for 30 min, the sections were washed with TBS 3 times for 5 min. Blocking was performed with sheep serum (20%, diluted with TBS) for 20 min at 37 °C. The sections were incubated overnight with primary antibody (See Supplementary Table S2) at 4 °C. A 1:200 dilution of secondary antibody (goat anti-rabbit) was added to each section and incubated for 45 min at RT and visualized with diaminobenzidine (DAB) color development. All experiments were repeated three times.

### Measurement of hydroxyproline content and serum biochemical assays

Hydroxyproline content was evaluated spectrophotometrically using a commercially available kit (Nanjing Jiancheng Bioengineering institute, Nanjing, China) according to the manufacturer’s instructions. Serum biochemical parameters, such as serum content of total protein and albumin, levels of AST, ALT, γ-GT and AKP were evaluated with commercially available kits (Nanjing Jiancheng Bioengineering institute, Nanjing, China) according to the manufacturers’ instructions. All assays were repeated three times.

### Cell culture

HSC-T6, an immortalized rat liver stellate cell line established by Dr. Scott L. Friedman, was a generous gift from his student, Professor Lie-Ming Xu (Shanghai University of Traditional Chinese Medicine, Shanghai). The human LX-2 hepatic stellate cell line was purchased from XiangYa Central Experiment Laboratory of Central South University (the same source as above). The two cell lines were cultured in DMEM supplemented with 10% fetal bovine serum (FBS) and 1% penicillin/streptomycin. All cells were cultured at 37 °C with 5% CO_2_.

### Construction of C/EBP-α mutant expression plasmids

The C/EBP-α mutant expression plasmids, which included the C/EBP-α-273K/R, C/EBP-α-275K/R and C/EBP-α-276K/R mutations, were constructed and confirmed by Hanbio Biotechnology Co., Ltd, Shanghai, China.

### Transient transfection

HEK293T cells were depleted of serum and incubated with antibiotic-free DMEM for 24 hrs before transfection. The cells were incubated with 1 μg of C/EBP-α mutant expression plasmid (or control plasmid) and 3 μl of Lipofectamine. Six hours after transfection, the cells were washed three times with phosphate-buffered saline and subsequently incubated in DMEM containing 10% fetal calf serum. Immunofluorescent images confirmed the transfection efficiency 24 hours after transfection (See Supplementary Fig. S4). Cells were harvested 24 hours after transfection and used in the following experiments. The experiments were repeated three times.

### Immunoprecipitation

Total protein was isolated from HSC-T6 and LX-2 cells, and incubated with a polyclonal rabbit anti-C/EBP-α antibody (sc-61, Santa Cruz) or a polyclonal rabbit anti-acetylated-Lysine antibody (#9441, Cell Signaling Technology) for 4 hrs at 4 °C. Non-specific rabbit IgG antibody (sc-2027, Santa Cruz) was used as a control. Anti-C/EBP-α antibody was used with 2 μg per 500 μg of total protein. Anti-acetylated-Lysine antibody was used with dilution of 1:100. Non-specific rabbit IgG antibody was used with 0.2 μg per 500 μl of lysate. Then, 30 μl of protein A/G-linked agarose beads (Beyotime, Jiangsu, China) were added. The mixture was incubated with rotation overnight at 4 °C. The beads were then washed three times with lysis buffer. The precipitated beads were collected, added with SDS buffer, and boiled at 100 °C for 5 min. The resulting immune precipitated protein product was evaluated by Western blot analysis. The experiments were repeated three times.

### Western blot analysis

HSC-T6 and LX-2 cells were collected, washed in cold phosphate-buffered saline (PBS), and lysed in RIPA buffer containing protease for 45 min on ice. Cell lysates were centrifuged at 13,000 g for 30 min, and proteins in the supernatant were quantified using a BCA protein assay kit. Total proteins were denatured in sodium dodecyl sulfate (SDS)-containing sample buffer, applied to a 10–12% SDS–polyacrylamide gel, then transferred to nitrocellulose membranes after electrophoresis. Proteins were detected with primary and secondary antibodies. The application of primary antibodies detailed in Supplementary Table S2. Secondary antibody (goat anti-rabbit and goat anti-mouse) was used with dilution of 1:1000. Protein bands were visualized using an ECL assay kit. All western blot analyses were repeated three times.

### Terminal deoxynucleotidyltransferase (TdT)-mediated dUTP nick end-labeling (TUNEL) assay

The TUNEL assay was used for the detection of cells undergoing apoptosis. We used a one-step TUNEL fluorescent kit (Beyotime, Jiangsu, China) to perform this experiment. The staining of the cells was performed according to the manufacturer’s recommended procedure. The TUNEL-positive cells were imaged under a fluorescence microscope (Nikon 80i, Tokyo, Japan) and quantified as the number of green spots in each photograph. The experiments were repeated three times.

### Flow cytometric analysis

The detection of apoptosis in HSCs was performed with an annexin V-FITC/PI kit (Beyotime, Jiangsu, China). Cells were harvested, washed twice with ice-cold PBS, and resuspended in 1 ml of binding buffer. The resuspended cells in 100 μl were transferred to a 1.5 ml EP tube, to which 5 μl of annexin V-FITC and 5 μl of PI were added. The mixture was gently vortexed and incubated in the dark at RT for 15 min. Binding buffer (400 μl) was then added, and flow cytometry was conducted to test the levels of apoptotic cells. The experiments were repeated three times.

### Isolation and culture of rat HSCs

Ten normal male Sprague–Dawley rats (body weight about 400 g) were used in this experiment. Animal handling was performed in accordance with the NIH Guidelines for the Care and Use of Laboratory Animals, and the protocol was approved by Animal Ethic Committee of Fudan University School of Basic Medical Sciences. Rat HSCs were isolated through a two-step digestion process. This was performed as described previously^5^. Primary cultured HSCs were used within 10 days of seeding in the following experiments.

### Statistical analysis

All experiments were repeated independently for three times. Experimental differences were analyzed by SPSS software (version 19.0) using Student’s t test and one-way analyses of variance tests (ANOVA) followed by LSD or Dunnett T3 tests. Ishak scores between different treatments were assessed by a Kruskal-Wallis test followed by post-hoc Dunn-Holland-Wolfe analysis. A *p* value of less than 0.05 was considered statistically significant.

### Data availability

The datasets generated during and/or analysed during the current study are available from the corresponding author on reasonable request.

All data generated or analysed during this study are included in this published article (and its Supplementary Information files).

## Electronic supplementary material

Supplementary information
