# Supplementary material for: Trichostatin A inhibits the activation of Hepatic stellate cells by Increasing C/EBP-α Acetylation in vivo and in vitro
Source: Sci Rep. 2018 Mar 13;8:4395. doi: 10.1038/s41598-018-22662-6 (PMC5849734; doi:10.1038/s41598-018-22662-6)
Supplement: Supplementary file 1 — Supplementary information [file 41598_2018_22662_MOESM1_ESM.pdf]

Trichostatin A inhibits the activation of Hepatic stellate cells by Increasing C/EBP-α Acetylation in vivo and in vitro

Di Ding[1,+], Lin-Lin Chen[2,+], Ying-Zhen Zhai[1], Chen-Jian Hou[1], Li-Li Tao[3], Shu-Han Lu[4], Jian Wu[5,6,*], Xiu-Ping Liu[1,2,*]

[1]Dept. of Pathology, School of Basic Medical Sciences, Fudan University, Shanghai 200032, China.

[2]Dept. of Pathology, The Fifth People's Hospital, Fudan University, Shanghai 200040, China.

[3]Dept. of Pathology, Peking University, Shenzhen Hospital, Shenzhen 518036, China.

[4]Dept. of Nutrition, University of California at Davis, Davis, California, USA.

[5]Dept. of Medical Microbiology, Key Laboratory of Molecular Virology, School of Basic Medical Sciences, Fudan University, Shanghai 200032, China.

[6]Shanghai Institute of Liver Disease, Fudan University, Shanghai 200032, China.

[+]these authors contributed equally to this work

*corresponding authors

Corresponding authors:

Xiu-Ping Liu, MD, PhD
Professor of Pathology
Dept. of Pathology, School of Basic Medical Sciences, Fudan University, 138 Yixue Yuan Road, Shanghai 200032, China.
Tel. +86-21-54237528
Fax: +86-21-54237596
Email: xpliu1228@fudan.edu.cn

Jian Wu, MD, PhD
Dept. of Medical Microbiology, Key Laboratory of Molecular Virology, School of Basic Medical Sciences, Fudan University, 138 Yixue Yuan Road, Shanghai 200032, China.
Tel. +86-21-54237705
Fax: +86-21-64227201
E-mail: jian.wu@fudan.edu.cn

Supplementary information

Score	Description
0	No fibrosis
1	Fibrosis expansion of some portal areas, with or without short fibrous septa
2	Fibrosis expansion of most portal areas, with or without short fibrous septa
3	Fibrosis expansion of most portal areas, with occasional portal to portal bridging
4	Fibrosis expansion of most portal areas, with marked portal to portal bridging as well as portal areas to central bridging
5	Marked bridging with occasional nodules
6	Probable to definite cirrhosis

Supplementary Table S1. Ishak scoring criteria.

Antibody	Manufacturer	Catalog number	Antibody species	Dilution
Anti-C/EBP-α	Santa Cruz	sc-61	Rabbit polyclonal antibody	1:100 for IHC<br>1:300 for WB
Anti-α-SMA	Abcam	ab124964	Rabbit monoclonal antibody	1:1000 for IHC<br>1:2000 for WB
Anti-α-SMA	Santa Cruz	sc-53142	mouse monoclonal antibody	1:50 for IF
Anti-collagen type I	Abcam	ab21286	Rabbit polyclonal antibody	1:200 for IHC<br>1:1000 for WB
Anti-acetylated-Lysine	Cell Signaling Technology	#9441	Rabbit polyclonal antibody	1:1000 for WB
Anti-ubiquitin	Sigma-Aldrich	U5379	Rabbit polyclonal antibody	1:100 for WB
Anti-caspase3	Cell Signaling Technology	#9662	Rabbit polyclonal antibody	1:1000 for WB
Anti-GAPDH	Santa Cruz	sc-166574	Mouse monoclonal antibody	1:1000 for WB

Supplementary Table S2. The manufacturer, catalog number, species, and dilution of antibody.

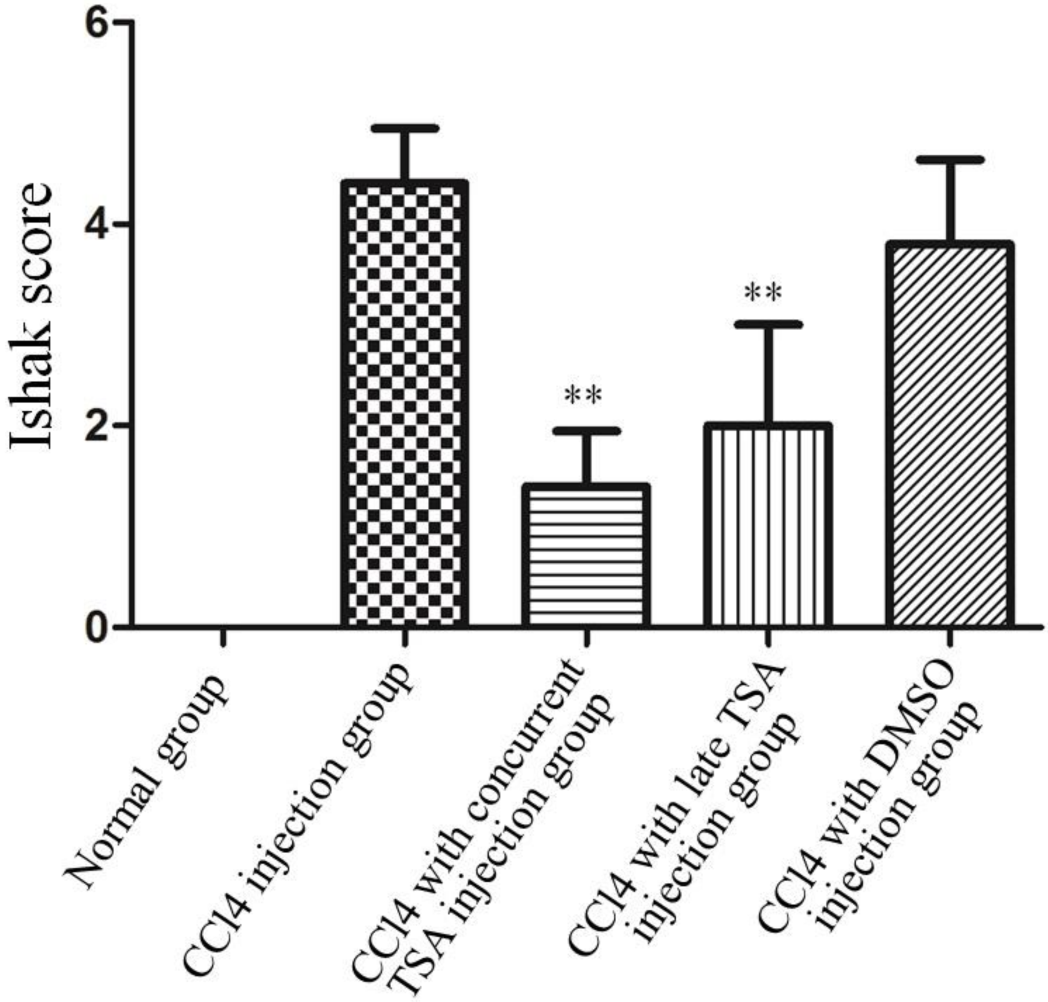

Supplementary Figure S1 The Ishak semi-quantitative scores of different groups. Sirius red staining of liver sections were scored by the method of Ishak (n = 6 for all groups). **p<0.01 compared to CCl_4 injection group.

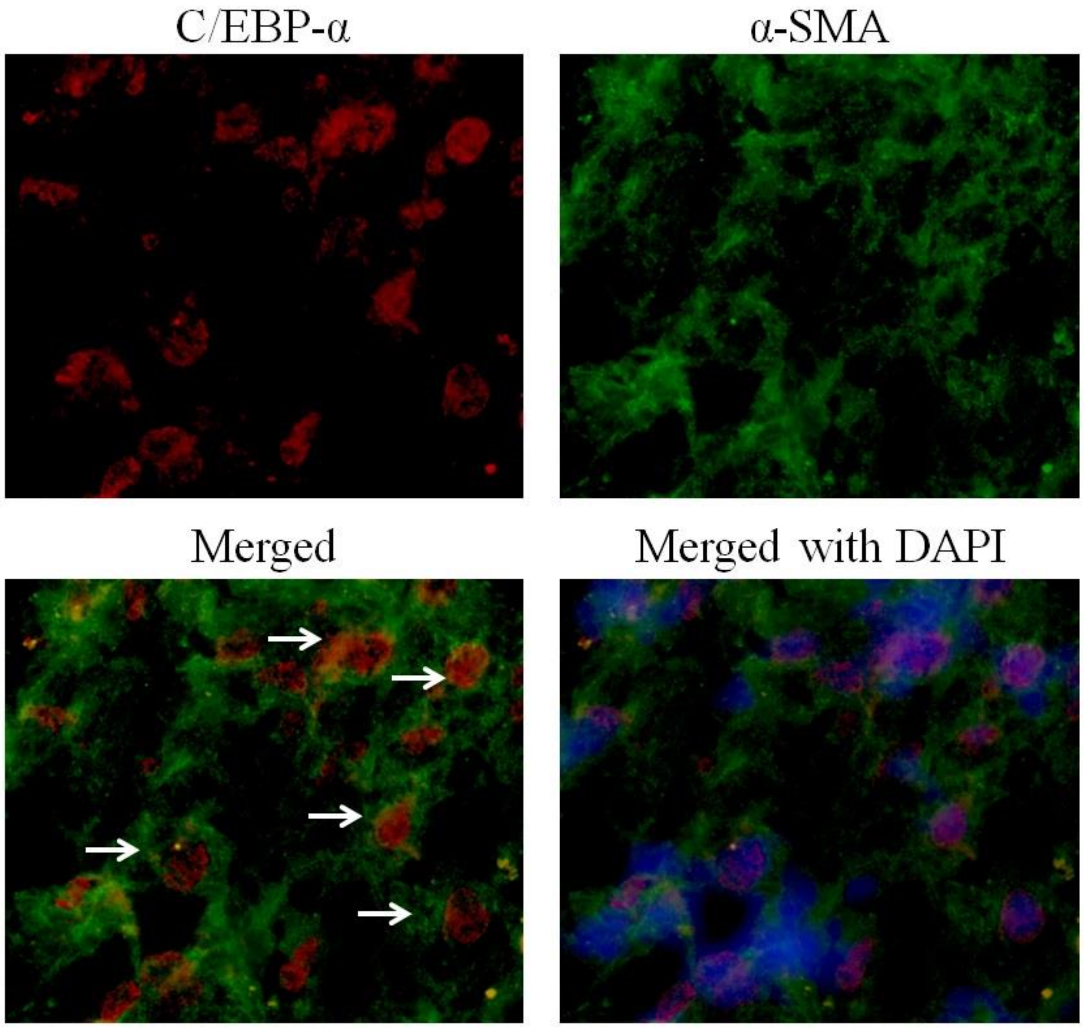

Supplementary Figure S2 Co-localization of C/EBP-α (red) with α-SMA (green) in liver by immunofluorescent staining in mice treated with CCl_4 plus TSA group. The merged image indicates the location of C/EBP-α in hepatic stellate cells (magnification, ×600). Arrows indicate the positive red C/EBP-α staining in the nucleus, whereas, green α-SMA in the cytosolic compartment.

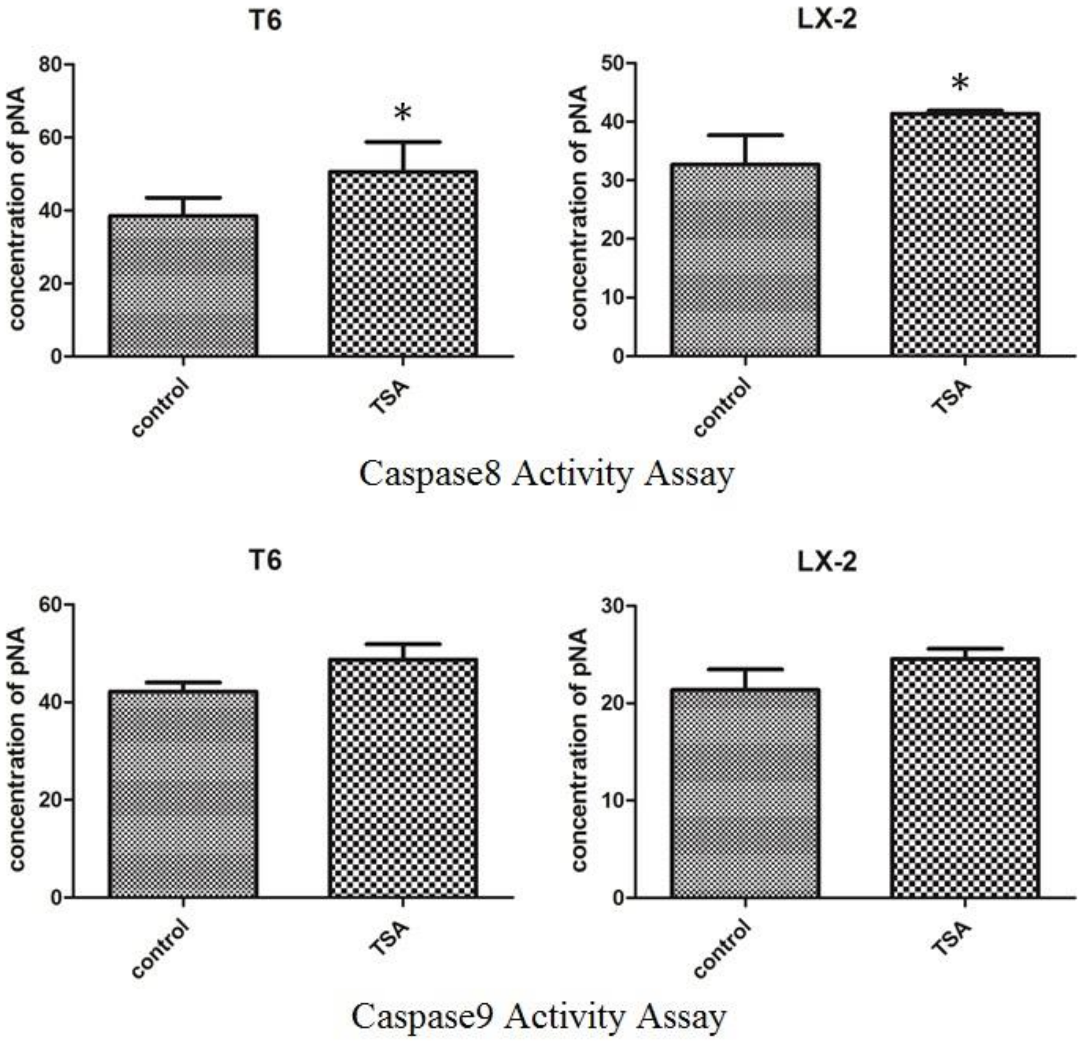

Supplementary Figure S3 The activity of both caspase-8 and caspase-9 were increased; however, caspase-9 activity did not reach statistical significance.

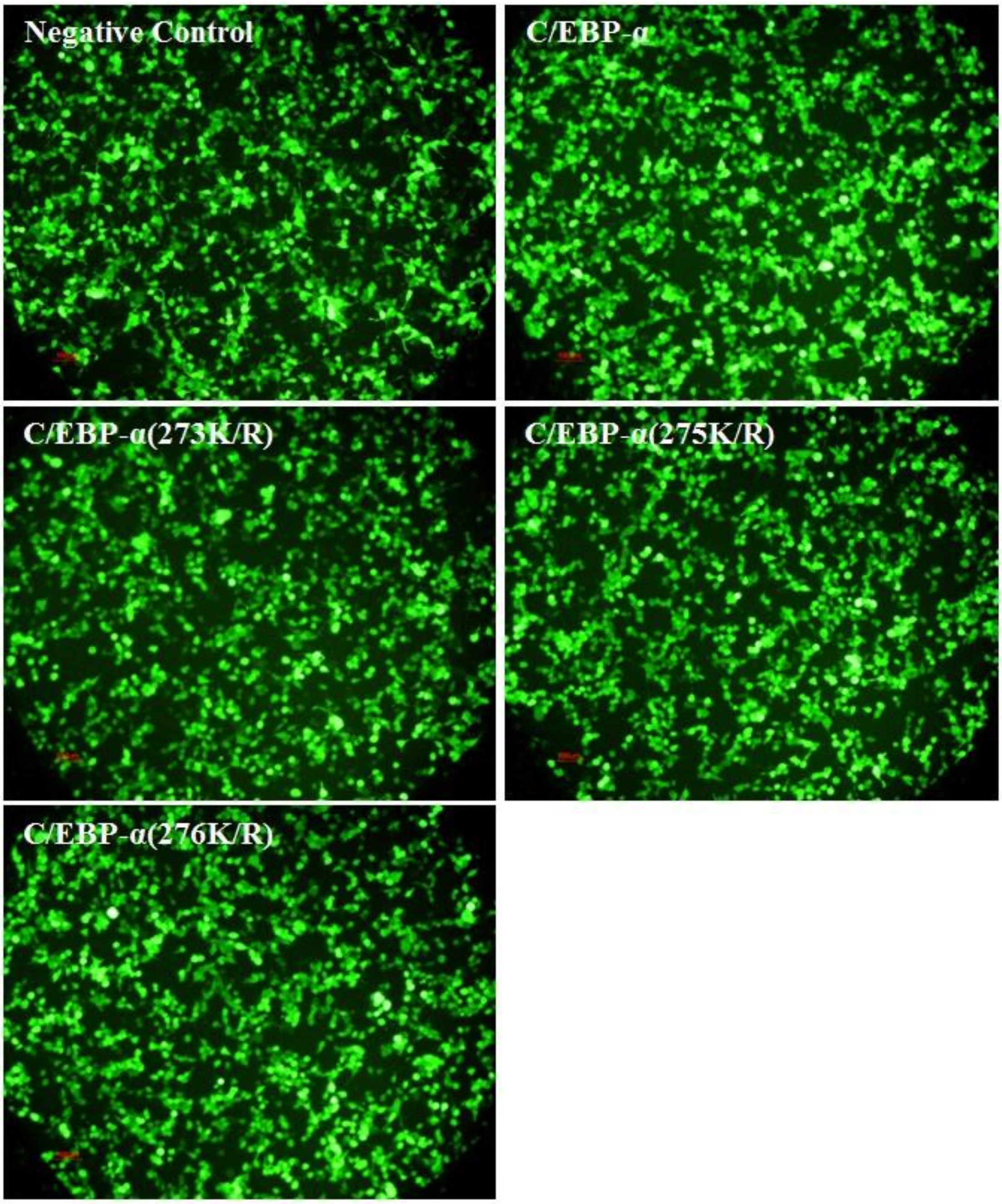

Supplementary Figure S4 Immunofluorescent images of C/EBP-α showed its transfection efficiency.

Figure 1

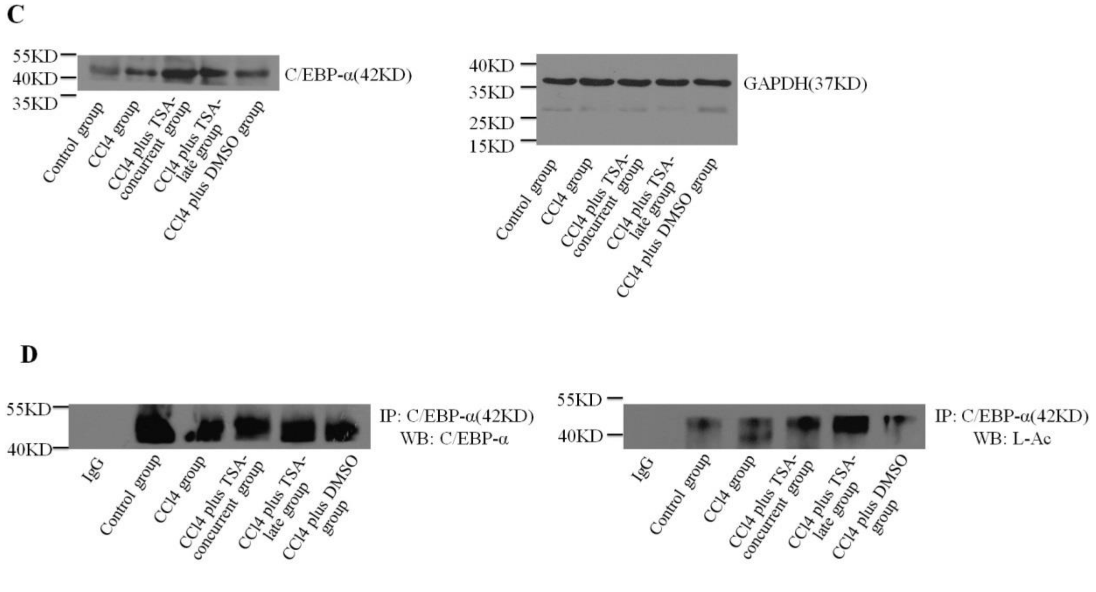
C
55KD
40KD
35KD
C/EBP-α(42KD)
Control group
CCl4 group
CCl4 plus TSA-concurrent group
CCl4 plus TSA-late group
CCl4 plus DMSO group
40KD
35KD
25KD
15KD
GAPDH(37KD)
D
55KD
40KD
IP: C/EBP-α(42KD)
WB: C/EBP-α
IgG
55KD
40KD
IP: C/EBP-α(42KD)
WB: L-Ac

Figure 2

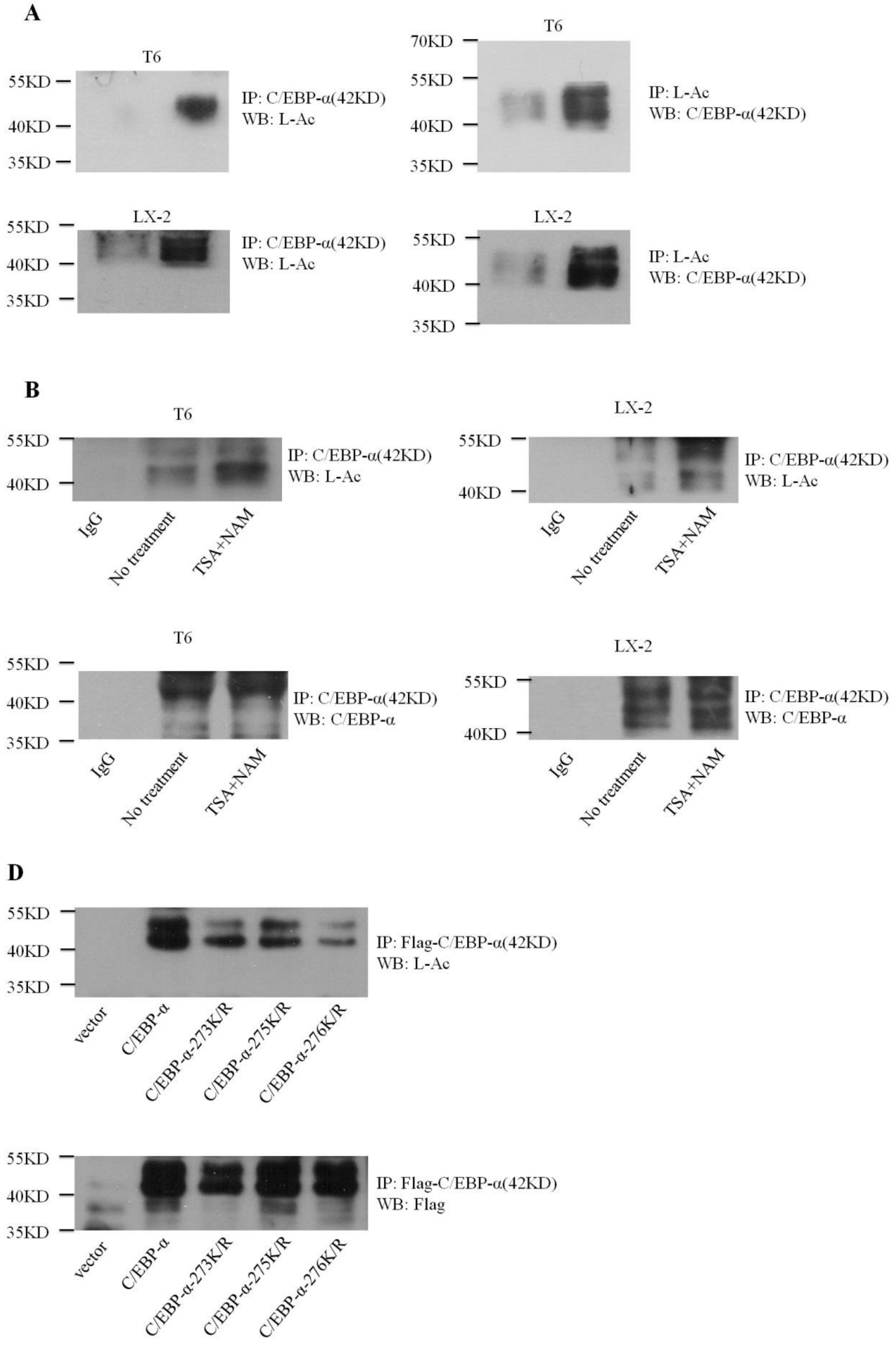

A
T6
55KD
40KD
35KD
IP: C/EBP-α(42KD)
WB: L-Ac
T6
70KD
55KD
40KD
35KD
IP: L-Ac
WB: C/EBP-α(42KD)
LX-2
55KD
40KD
35KD
IP: C/EBP-α(42KD)
WB: L-Ac
LX-2
55KD
40KD
35KD
IP: L-Ac
WB: C/EBP-α(42KD)
B
T6
55KD
40KD
IP: C/EBP-α(42KD)
WB: L-Ac
IgG
No treatment
TSA+NAM
LX-2
55KD
40KD
IP: C/EBP-α(42KD)
WB: L-Ac
IgG
No treatment
TSA+NAM
T6
55KD
40KD
35KD
IP: C/EBP-α(42KD)
WB: C/EBP-α
IgG
No treatment
TSA+NAM
LX-2
55KD
40KD
IP: C/EBP-α(42KD)
WB: C/EBP-α
IgG
No treatment
TSA+NAM
D
55KD
40KD
35KD
IP: Flag-C/EBP-α(42KD)
WB: L-Ac
vector
C/EBP-α
C/EBP-α-273K/R
C/EBP-α-275K/R
C/EBP-α-276K/R
55KD
40KD
35KD
IP: Flag-C/EBP-α(42KD)
WB: Flag
vector
C/EBP-α
C/EBP-α-273K/R
C/EBP-α-275K/R
C/EBP-α-276K/R

Figure 3

A

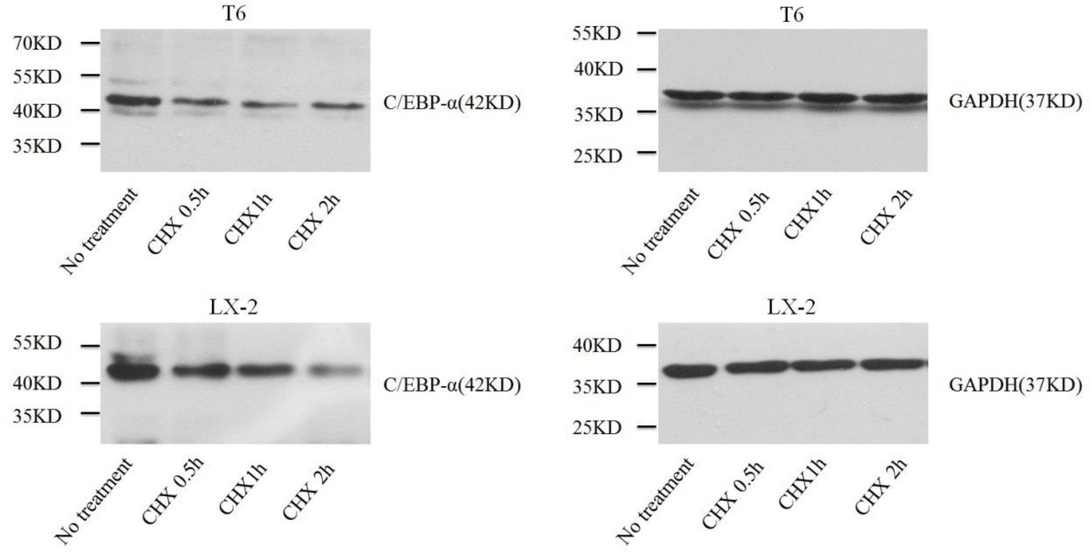
T6
70KD
55KD
40KD
35KD
C/EBP-α(42KD)
No treatment
CHX 0.5h
CHX1h
CHX 2h
T6
55KD
40KD
35KD
25KD
GAPDH(37KD)
No treatment
CHX 0.5h
CHX1h
CHX 2h
LX-2
55KD
40KD
35KD
C/EBP-α(42KD)
No treatment
CHX 0.5h
CHX1h
CHX 2h
LX-2
40KD
35KD
25KD
GAPDH(37KD)
No treatment
CHX 0.5h
CHX1h
CHX 2h

B

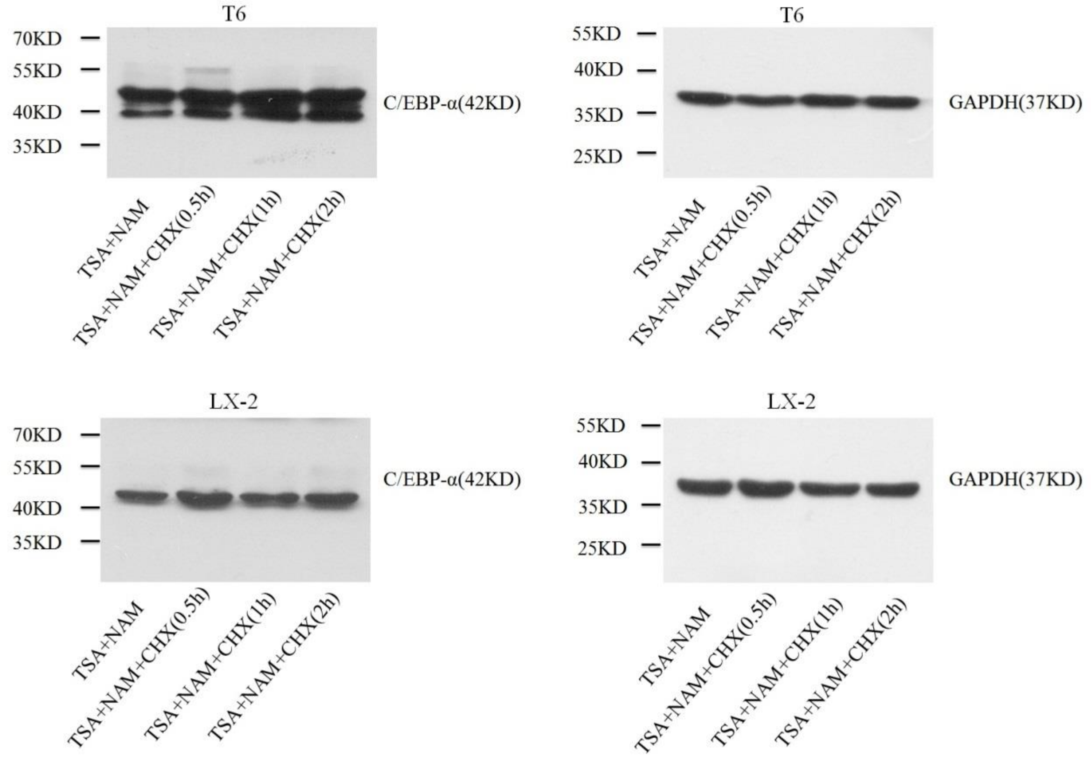
T6
70KD
55KD
40KD
35KD
C/EBP-α(42KD)
TSA+NAM
TSA+NAM+CHX(0.5h)
TSA+NAM+CHX(1h)
TSA+NAM+CHX(2h)
T6
55KD
40KD
35KD
25KD
GAPDH(37KD)
TSA+NAM
TSA+NAM+CHX(0.5h)
TSA+NAM+CHX(1h)
TSA+NAM+CHX(2h)
LX-2
70KD
55KD
40KD
35KD
C/EBP-α(42KD)
TSA+NAM
TSA+NAM+CHX(0.5h)
TSA+NAM+CHX(1h)
TSA+NAM+CHX(2h)
LX-2
55KD
40KD
35KD
25KD
GAPDH(37KD)
TSA+NAM
TSA+NAM+CHX(0.5h)
TSA+NAM+CHX(1h)
TSA+NAM+CHX(2h)

C

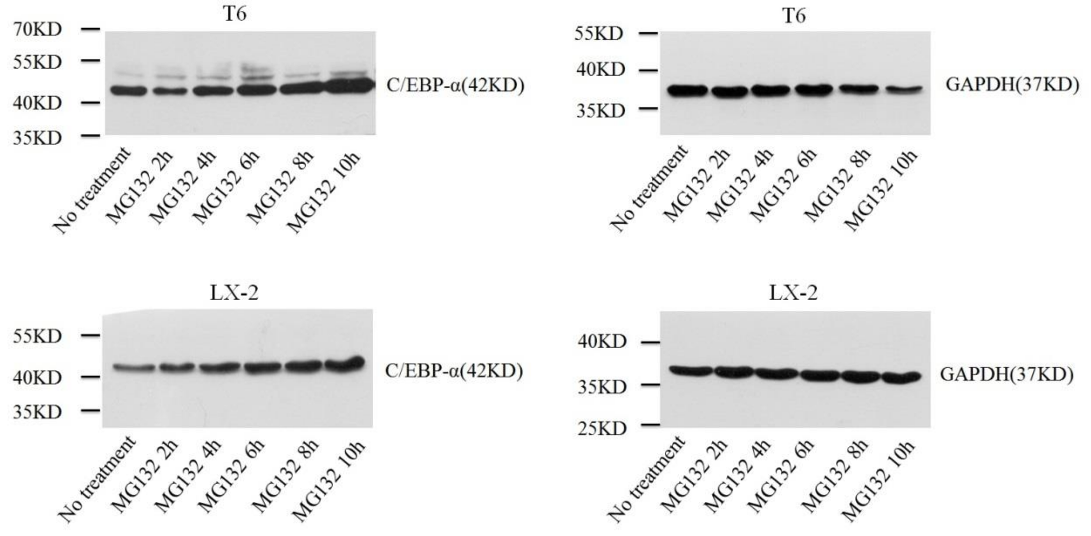
T6
70KD
55KD
40KD
35KD
C/EBP-α(42KD)
No treatment
MG132 2h
MG132 4h
MG132 6h
MG132 8h
MG132 10h
T6
55KD
40KD
35KD
GAPDH(37KD)
LX-2
55KD
40KD
35KD
C/EBP-α(42KD)
LX-2
40KD
35KD
25KD
GAPDH(37KD)

D

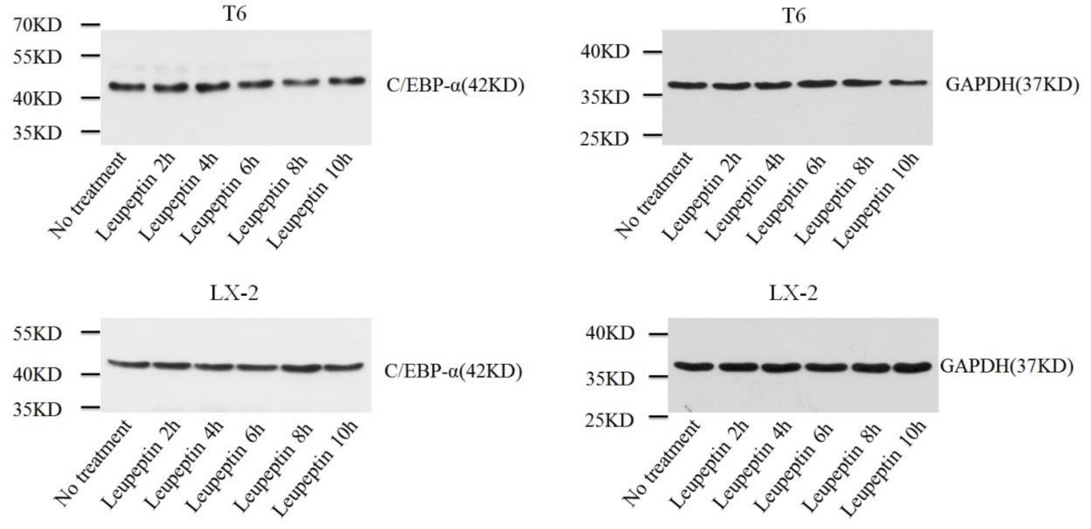
T6
70KD
55KD
40KD
35KD
C/EBP-α(42KD)
No treatment
Leupeptin 2h
Leupeptin 4h
Leupeptin 6h
Leupeptin 8h
Leupeptin 10h
T6
40KD
35KD
25KD
GAPDH(37KD)
LX-2
55KD
40KD
35KD
C/EBP-α(42KD)
LX-2
40KD
35KD
25KD
GAPDH(37KD)

E

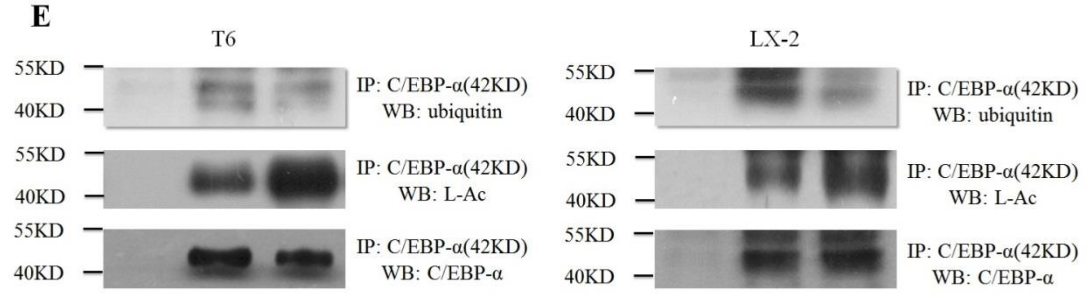
T6
55KD
40KD
IP: C/EBP-α(42KD)
WB: ubiquitin
IP: C/EBP-α(42KD)
WB: L-Ac
IP: C/EBP-α(42KD)
WB: C/EBP-α
LX-2
55KD
40KD
IP: C/EBP-α(42KD)
WB: ubiquitin
IP: C/EBP-α(42KD)
WB: L-Ac
IP: C/EBP-α(42KD)
WB: C/EBP-α

Figure 4

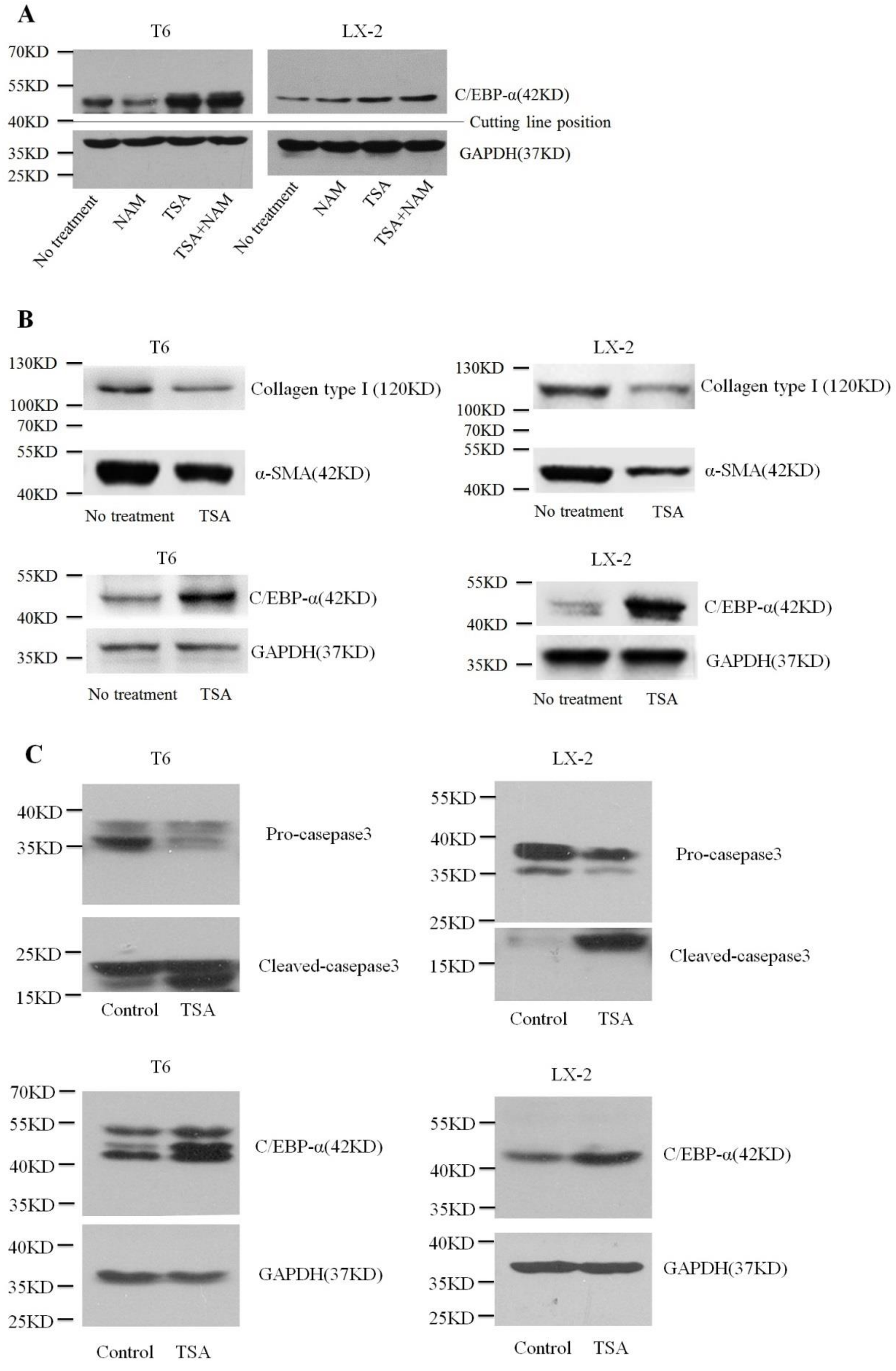
A
T6
LX-2
70KD
55KD
40KD
35KD
25KD
C/EBP-α(42KD)
Cutting line position
GAPDH(37KD)
No treatment
NAM
TSA
TSA+NAM
B
130KD
100KD
Collagen type I (120KD)
α-SMA(42KD)
C
Pro-casepase3
Cleaved-casepase3
15KD
Control

Figure 5

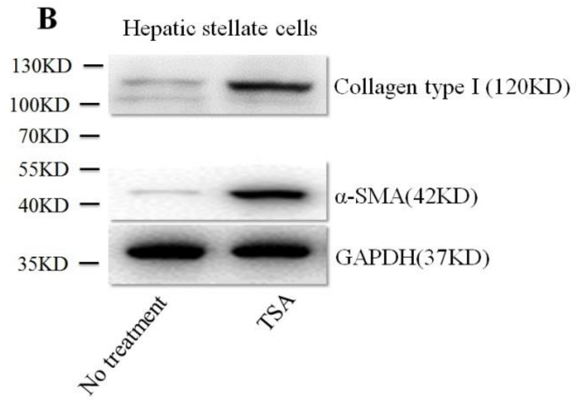
B
Hepatic stellate cells
130KD
100KD
70KD
55KD
40KD
35KD
Collagen type I (120KD)
α-SMA(42KD)
GAPDH(37KD)
No treatment
TSA

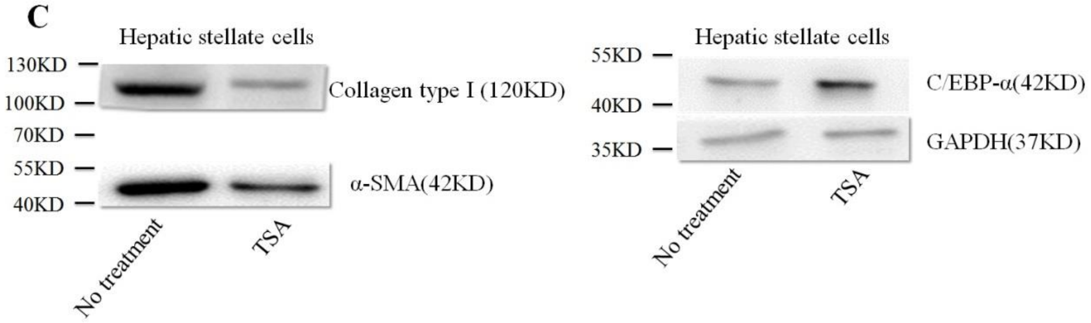
C
Hepatic stellate cells
130KD
100KD
70KD
55KD
40KD
Collagen type I (120KD)
α-SMA(42KD)
No treatment
TSA
Hepatic stellate cells
55KD
40KD
35KD
C/EBP-α(42KD)
GAPDH(37KD)
No treatment
TSA
